# Fabrication of Poly(ethylene furanoate)/Silver and Titanium Dioxide Nanocomposites with Improved Thermal and Antimicrobial Properties

**DOI:** 10.3390/ma17071606

**Published:** 2024-04-01

**Authors:** Johan Stanley, Eleftheria Xanthopoulou, Lidija Fras Zemljič, Panagiotis A. Klonos, Apostolos Kyritsis, Dimitra A. Lambropoulou, Dimitrios N. Bikiaris

**Affiliations:** 1 Laboratory of Chemistry and Technology of Polymers and Colors, Department of Chemistry, Aristotle University of Thessaloniki, GR-54124 Thessaloniki, Greece; johansta@chem.auth.gr (J.S.); elefthxanthopoulou@gmail.com (E.X.); 2 Faculty of Mechanical Engineering, University of Maribor, SI-2000 Maribor, Slovenia; lidija.fras@um.si; 3 Department of Physics, National Technical University of Athens, Zografou Campus, GR-15780 Athens, Greece; pklonos@central.ntua.gr (P.A.K.); akyrits@central.ntua.gr (A.K.); 4 Laboratory of Environmental Pollution Control, Department of Chemistry, Aristotle University of Thessaloniki, GR-54124 Thessaloniki, Greece; dlambro@chem.auth.gr; 5 Center for Interdisciplinary Research and Innovation (CIRI-AUTH), Balkan Center, GR-57001 Thessaloniki, Greece

**Keywords:** active agents, antimicrobial studies, biobased polymers, crystallinity, poly(ethylene 2,5-furandicarboxylate)

## Abstract

Poly(ethylene furanoate) (PEF)-based nanocomposites were fabricated with silver (Ag) and titanium dioxide (TiO_2_) nanoparticles by the in-situ polymerization method. The importance of this research work is to extend the usage of PEF-based nanocomposites with improved material properties. The PEF-Ag and PEF-TiO_2_ nanocomposites showed a significant improvement in color concentration, as determined by the color colorimeter. Scanning electron microscopy (SEM) photographs revealed the appearance of small aggregates on the surface of nanocomposites. According to crystallinity investigations, neat PEF and nanocomposites exhibit crystalline fraction between 0–6%, whereas annealed samples showed a degree of crystallinity value above 25%. Combining the structural and molecular dynamics observations from broadband dielectric spectroscopy (BDS) measurements found strong interactions between polymer chains and nanoparticles. Contact angle results exhibited a decrease in the wetting angle of nanocomposites compared to neat PEF. Finally, antimicrobial studies have been conducted, reporting a significant rise in inhibition of over 15% for both nanocomposite films against gram-positive and gram-negative bacteria. From the overall results, the synthesized PEF-based nanocomposites with enhanced thermal and antimicrobial properties may be optimized and utilized for the secondary packaging (unintended food-contact) materials.

## 1. Introduction

Biobased polymers have gained considerable interest in food packaging applications as they eliminate the danger of toxicity to consumers [1]. Despite the poor mechanical properties, biobased polymers are generally considered an organic matrix for incorporating antimicrobial agents [2]. Poly(L-lactic acid) (PLLA) is one of the most widely used and promising bio-based polymers that have already been industrialized. Adding various active agents, such as chitosan and essential basil oil, has been extensively studied to improve the thermal, mechanical, antioxidant, and barrier properties of the composites [3]. Also, polyethylene glycol (PEG)’s solvatable polymer backbone, neutral surface charge, and chemical stability make it one of the most adaptable molecular stabilizers of inorganic nanoparticles [4]. In our present work, specifically, a bio-based polymer, 100% fully recyclable poly(ethylene 2,5-furandicarboxylate) (PEF), offers higher mechanical strength and stiffness than its fossil-based poly(ethylene terephthalate) (PET) counterpart [5]. In addition, PEF considerably reduces greenhouse gas emissions and non-renewable energy compared to the widely used PET [6]. Recently, few studies have been carried out to synthesize PEF-based nanocomposite films to enhance the antimicrobial nature and barrier properties of PEF-targeting packaging applications [7,8]. Also, our previous work has been focused on the synthesis of PEF-based nanocomposites with cerium bioglass, zinc oxide, and zirconium dioxide as active agents with enhanced antimicrobial resistance for food packaging materials [9].

Active packaging systems possess an impressive surge of interest from the packaging sector in improving shelf life, food recalls, and foodborne illness outbreaks [10]. These systems can be prepared using several techniques, including incorporating active agents, coatings, immobilization, or surface modification onto packaging materials [11]. Nanoparticles have been widely used in packaging materials, providing better preservation and quality maintenance of food products than conventional packaging materials. Furthermore, the addition of additives modifies the properties (physical and mechanical) of the packaging polymer, offering enhanced strength, flexibility, durability, and barrier properties [12]. Since the proliferation of pathogenic and/or spoilage microorganisms was the main factor contributing to food spoiling, antimicrobial agents are the active agent classes with the most significant number of commercial products [11,13,14].

Metals and metal oxide nanoparticles, such as Ag, ZnO, Cu, CuO, CuS, MgO, Se, Pd, Fe, SiO_2_, and TiO_2_, are commonly utilized antimicrobial agents in packaging applications because they generally exhibit the highest antibacterial activity [15]. Inorganic nanoparticles typically contain reactive sites and polar functional groups that interact differently with liquids than the polymer matrix. These interactions can cause a shift towards lower contact angles. Further, the presence of nanoparticles within the polymer matrix can enhance the overall surface roughness of the material; a higher surface roughness generally correlates with lower contact angles due to the increased likelihood of liquid penetration into the irregular surface features [16]. Mendes et al. reported that the incorporation of BaTiO_3_ particles in the poly(vinylidene fluoride) (PVDF) polymer matrix enhances the thermal stability of the composite; in contrast, the degree of crystallinity decreases with increasing filler content [17].

In this context, silver nanoparticles (AgNPs) appeared to be a choice for biopolymer-nanoparticle composite material used for packaging materials due to their high antimicrobial and antioxidant activity [18]. The plant-based production of AgNPs was simple, non-toxic, cost-effective, eco-friendly, and potent against fungi and bacteria [19]. Moreover, at low concentrations, AgNPs are non-toxic to the human body [20,21]. Azlin-Hasim et al. studied the microbiological quality of low-density polyethylene films (LDPE) containing different concentrations of AgNPs for modified atmosphere packaging. The results indicated that regardless of AgNP concentrations, all nanocomposite films showed extended shelf life and oxidative stability [22].

Titanium dioxide nanoparticles (TNPs) potentially develop compelling antimicrobial films to extend shelf life and ensure safety in packaging industries. Indeed, TiO_2_ is inert, inexpensive, and acts as a UV light filter [23]. At the nanoscale level, the particle size of TiO_2_ decreases, and surface area increases dramatically, which is the desired feature for photocatalytic activity to inactivate the broad spectrum of microorganisms [24]. Siripatrawan et al. developed ethylene scavenging and antimicrobial films from nanocomposite chitosan and different concentrations of TiO_2_ nanoparticles for food packaging applications. The results showed that chitosan film containing 1 wt.% of TiO_2_ was found to be optimal and exhibited antimicrobial activity against Gram-positive bacteria, Gram-negative bacteria, and fungi [25].

Herein, PEF-based nanocomposites were synthesized using AgNPs and TNPs (particle size less than 100 nm) to improve the thermal properties and bacterial inhibition percentage of the nanocomposites. It is the first time that such nanocomposites have been fabricated using specific active agents via the in-situ polymerization method to disperse active agents uniformly into the PEF matrix and to expand their usage in packaging applications. Furthermore, synthesized nanocomposites have been extensively characterized regarding their structural and physico-chemical properties, crystallinity, molecular mobility, hydrophilicity, and antimicrobial activity. The developed materials were examined in terms of long-term performance under simulated packaging conditions.

## 2. Materials and Methods

### 2.1. Materials

2,5- furan dicarboxylic acid (BioFDCA X000230-2003) was purchased from Corbion, (Gorinchem, The Netherlands). Ethylene glycol (anhydrous, 99.8%) and antimony trioxide were purchased from Aldrich Co., (London, UK). Ag and TiO_2_ were purchased from Alfa Aesar (Ward Hill, MA, USA), and Sigma Aldrich, (St. Louis, MO, USA), respectively. The PEF polyester and PEF-based nanocomposites were synthesized as reported in our previous studies [9].

### 2.2. Characterization

#### 2.2.1. Intrinsic Viscosity ([*η*]) and Molecular Weight (*M_n_*) Determination

A Ubbelohde viscometer (Schott Gerate GMBH, Hofheim, Germany) has been utilized to determine the [*η*] of PEF and respective nanocomposites with (60/40, *w*/*w*) of phenol/1,1,2,2-tetrachloroethane at the temperature of 30 °C. The Solomon–Ciuta equation was used to calculate the [*η*] value of each sample (1):

(1) $$\left[\eta \right]=\frac{{\left[2\{\frac{t}{t_{0}}-\ln\left(\frac{t}{t_{0}}\right)-1\}\right]}^{\frac{1}{2}}}{c}$$

while *c* is the solution concentration, *t*_0_ is the flow time of pure solvent, and *t* is the flow time of solution.

The *M_n_* of the samples was calculated using the Berkowitz Equation (2) [9]:

(2) $$M\bar{n}=3.29\times 10^{4}\;{\left[\eta \right]}^{1.54}$$

#### 2.2.2. Color Analysis

Using an illuminant of D65 and a 10° standard observer, the color of the PEF and corresponding nanocomposites films was assessed using a Datacolor Spectraflash SF600 plus CT UV reflectance colorimeter (Datacolor, Marl, Germany), which included the specular component but excluded the UV component. The CIEL*a*b* color system was employed.

#### 2.2.3. Scanning Electron Microscopy (SEM)

A JEOL JMS 760F (Jeol, Freising, Germany) scanning microscope with an Oxford ISIS 300 energy dispersive X-ray microanalysis system was employed. In order to prevent charging under the electron beam, carbon black was applied to every surface during sample preparation.

#### 2.2.4. Attenuated Total Reflectance Fourier Transform Infrared Spectroscopy (ATR-FTIR)

An IRTracer-100 (Shimadzu, Tokyo, Japan) equipped with a QATR^TM^ 10 Single-Reflection ATR Accessory with a Diamond Crystal was used to record the samples’ ATR-FTIR spectra. In order to obtain the spectra, 16 co-added scans were performed from 450 cm^−1^ to 4000 cm^−1^ at a resolution of 2 cm^−1^. During this time, the baseline was adjusted and put into absorbance mode.

#### 2.2.5. Time-of-Flight Secondary Ion Mass Spectrometry (ToF-SIMS)

PEF-based nanocomposites were subjected to depth profiling and associated 3D imaging using a ToF-SIMS M6 device (IONTOF, Münster, Germany). The target current of a Bi_3_^+^ primary ion beam was set at 0.6 pA. A 5 keV gas cluster ion beam (GCIB) was employed for sputtering rastering over a 500 × 500 µm area. During the measurements, the flood cannon was in operation, the topographical mode was employed, a surface potential of 500 V was applied, and Ar gas flooding (5 × 10^−7^ torr) was turned on. For mass calibration, the known mass-to-charge (*m*/*z*) ratios of C^+^ (peak at *m*/*z* 12.00), C_2_H_5_^+^ (peak at *m*/*z* 27.02), and C_3_H_5_^+^ (peak at *m*/*z* 41.04) were utilized.

#### 2.2.6. Differential Scanning Calorimetry (DSC)

The thermal transitions were initially examined using TA Q200 DSC equipment (TA Instruments, New Castle, DE, USA), which was validated using sapphires for heat capacity and indium for temperature and enthalpy. Weighing between 6–7 mg, the fabricated samples were put in aluminum T_zero_ TA pans and exposed to temperature variations of 0 to 260 °C in a 99.9% pure nitrogen atmosphere. To eliminate any thermal history and provide the most excellent possible contact between the material being tested and the aluminum pan, the polymers were initially elevated to 260 °C. After that, two cooling–heating runs were performed to create amorphous and semicrystalline polymers, respectively. The initial run used a rapid cooling rate (scan 1, 100–110 °C/min, in the area of predicted crystallization), while the second used a typical cooling rate (scan 2, 20 °C/min). In conclusion, the subsequent heating scans were taken at a set heating rate of 10 °C/min.

To assess the weak polymer crystallinity in further detail, a PerkinElmer Pyris DSC-6 differential scanning calorimeter calibrated using pure indium and zinc standards was used. The temperature transitions of samples subjected to isothermal melt-crystallization annealing were measured using 5 ± 0.1 mg of samples enclosed in aluminum pans. Each experiment was carried out with a flow rate of 20 mL/min in a N_2_ environment. The following formula was used to calculate the degree of crystallinity (*X_c_*) (3):

(3) $${X_{c}}_{\;}(\%)=\left(\frac{\Delta H_{m}-\Delta H_{\mathrm{cc}}}{\left(1-w\right)\times \Delta H_{m}^{0}}\right)\times 100$$

where $\Delta H_{m}^{0}$ = 137 J/g, as reported by our team in our previous work for the melting heat of the 100% crystalline PEF.

#### 2.2.7. X-ray Diffraction (XRD)

Utilizing a MiniFlex II XRD equipment (Rigaku Co., Tokyo, Japan) with Cu Ka radiation (0.154 nm) and a scanning rate of 1 °/min spanning the 2*θ* range of 5° to 50°, the XRD spectra were recorded. The semicrystalline structure of every sample that had been melted and annealed (at 160 °C for an hour) at room temperature was examined. The percentage crystallinity was determined from the XRD graphs using the following Equation (4):

(4) $$X_{c}={\left(1+\frac{A_{am}}{A_{c}}\right)}^{-1}$$

*A_am_* is the area of the amorphous halo, and *A_c_* is the area of the crystalline peaks.

#### 2.2.8. Broadband Dielectric Spectroscopy (BDS)

BDS was used to study the molecular dynamics in a nitrogen environment, emphasizing segmental mobility. The setup for recording the data consisted of an Alpha frequency response analyzer (FRA) and a Novocontrol Quatro liquid nitrogen cryosystem (Novocontrol GmbH, Montabaur, Germany). Initial amorphous samples (melted and rapidly cooled) were utilized for the measurements, carried out in a sandwich-like capacitor with a diameter of 20 mm and an electrode spacing of around 1.2 mm (sample thickness). As a function of frequency in the range of 10^−1^ to 10^6^ Hz and temperature in the range of 120 °C to 150 °C, the complex dielectric permittivity, ε* = ε′ − i·ε″, is determined isothermally.

#### 2.2.9. Contact Angle (CA) Analysis

The contact angles were measured with a goniometer from DataPhysics (Filderstadt, Germany) using 3 μL of droplet volume and ultra-pure water (Millipore, Burlington, MA, USA). The values were determined at ambient temperature. Using the GraphPad Prism 6 program, a one-way ANOVA was implemented for the statistical analysis. A *p*-value of less than 0.05 demonstrated the statistically significant difference.

#### 2.2.10. Antibacterial Studies

Following the internal protocols of the Department of Microbiological Research, Center for Medical Microbiology of the National Laboratory for Health, Environment, and Food in Maribor, No. P96’ Biofilm production on various materials *Staphylococcus aureus*’ P90 (ISO22196) was employed to determine plastic surfaces’ microbiological activity/antimicrobial activity [26]. Neat PEF and PEF nanocomposite films were tested for their ability to inhibit the growth of *Escherichia coli* (DSM 1576) and *Staphylococcus aureus* (DSM 799) bacteria. To be more precise, 0.5 on the McFarland scale was used to inoculate neat PEF and PEF nanocomposite films of 10 mm × 10 mm with *Staphylococcus aureus* and *Escherichia coli*. For each type of bacteria, the test method was carried out three times (n = 3). A two-way ANOVA with repeated measurements was employed.

## 3. Results

### 3.1. Fabrication and Color Measurements of PEF and Respective Nanocomposites

PEF and respective nanocomposites were fabricated using the two-stage melt polycondensation method. Based on the literature, the type of nanoparticles used for synthesizing nanocomposites also has a minimal effect on influencing the *M_n_* of the nanocomposites [9]. The nanoparticles were incorporated at a low percentage (1 wt.%) taking into consideration the need to avoid agglomeration of particles in the polymer matrix. Siripatrawan et al. reported the direct correlation between filler content and porosity; also, a high-viscosity polymer matrix prevents dissemination between the particles, which causes void development and depletion of mechanical strength [25]. The PEF-Ag sample displays the highest intrinsic viscosity value between two nanocomposites, equal to 0.47 dL/g, as shown in Table 1. Kim et al. reported that the use of AgNPs (up to 6 wt.%) for fabricating biodegradable polylactide (PLA) nanofibers resulted in increased intrinsic viscosity values due to the corresponding increase in chain dimension, while further increase of the wt.% of AgNPs (9%) obstructed the chain dimension and resulted in a decreased intrinsic viscosity value [27]. The increase in *M_n_* of the PEF-Ag sample has been discussed in detail by applying dielectric spectroscopy. The *M_n_* values were calculated using the Berkowitz equation (Table 1).

**Figure 1:**
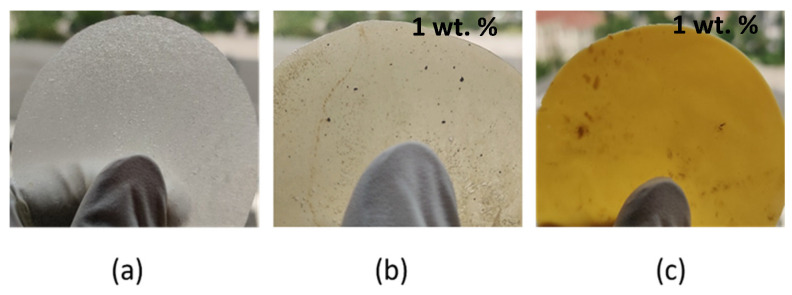
Film images of (**a**) neat PEF, (**b**) PEF-Ag, and (**c**) PEF-TiO_2_ nanocomposites.

PEF synthesized from FDCA is brighter due to the monomer’s high purity, as shown in Figure 1a. Also, the Sb_2_O_3_ catalyst used in the polycondensation step shows high activity and helps to produce polyester with low coloration [28]. The neat PEF displayed higher L* and lower K/S values, indicating that films are brighter and have low color concentration. Both nanocomposites showed an increase in color concentration after incorporating active agents, compared to neat PEF polyesters. The color calorimetric measurements were performed to measure the accurate color concentration of the nanocomposites, which are presented in Table 2.

The a* coordinate measures redness when a is positive or greenness when a is negative; the b* coordinate measures yellowness when b is positive or blueness when b is negative; C* represents chroma; H* represents hue angle; and the K/S fraction was calculated to determine the color concentration. The luminosity or brightness, which ranges from 0 (black) to 100 (white), is determined by the L* axis. The film images of neat PEF and respective nanocomposites are exhibited in Figure 1a–c. Specifically, the PEF-Ag nanocomposite color changes to a light brown (Figure 1b), indicating the presence of AgNPs **[29]**. PEF-TiO_2_ showed a decrease in L* and h° values due to the film’s low brightness and reduction in the film’s transparency, as seen in Figure 1c. According to Sani et al., TNPs in composite films block short-wavelength UV rays better than long-wavelength visible rays, preventing light transmission. As a result, the films’ transparency is reduced, but their UV-blocking qualities significantly rise [30]. This effect promotes the TNP composites as appealing materials for packaging applications.

### 3.2. SEM-EDX

**Figure 2:**
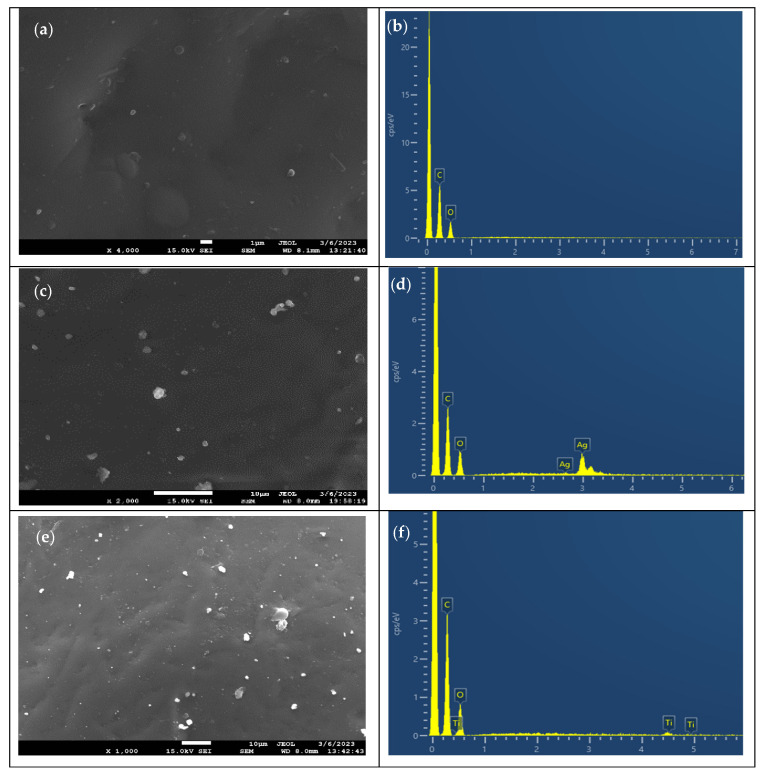
SEM micrographs of (**a**) neat PEF, (**c**) PEF-Ag, and (**e**) PEF-TiO_2_ nanocomposites. EDX spectrum of (**b**) neat PEF, (**d**) PEF-Ag, and (**f**) PEF-TiO_2_ nanocomposites. SEM micrographs of the neat PEF were obtained at a magnification of ×4000, PEF-Ag nanocomposites at ×2000, and PEF-TiO_2_ nanocomposites at ×1000.

The surface morphology and elemental content of PEF and respective nanocomposites were investigated using SEM-EDX analysis. Figure 2a shows that neat PEF exhibited a surface without many cracks or roughness. In contrast, the SEM images of PEF-based nanocomposites revealed the presence of small aggregates on the surface, as seen in Figure 2c,e. The fabrication of nanocomposites (with 1 wt.% of nanoparticles) by in-situ polymerization inhibits the agglomeration of particles by incorporating evenly distributed nanoparticles into the PEF matrix [31]. Based on the literature, an increase in the weight percentage of nanoparticles causes the particles to aggregate and form clusters in the polymer matrix, which alters the characteristics of nanocomposites [32].

Figure 2b,d,f show the EDX spectra of neat PEF and respective nanocomposites. The carbon atom is linked to the first strong peak at 0.2 keV, while the oxygen atom is linked to the second minor peak at 0.5 keV in every EDX profile. The appearance of new peaks in Figure 2d,f indicates the presence of AgNPs and TNPs in the polymeric matrix, respectively. Thus, EDX spectra analysis confirms that nanoparticles were successfully incorporated into the PEF matrix by in-situ polymerization method.

### 3.3. ATR-FTIR Spectroscopy

**Figure 3:**
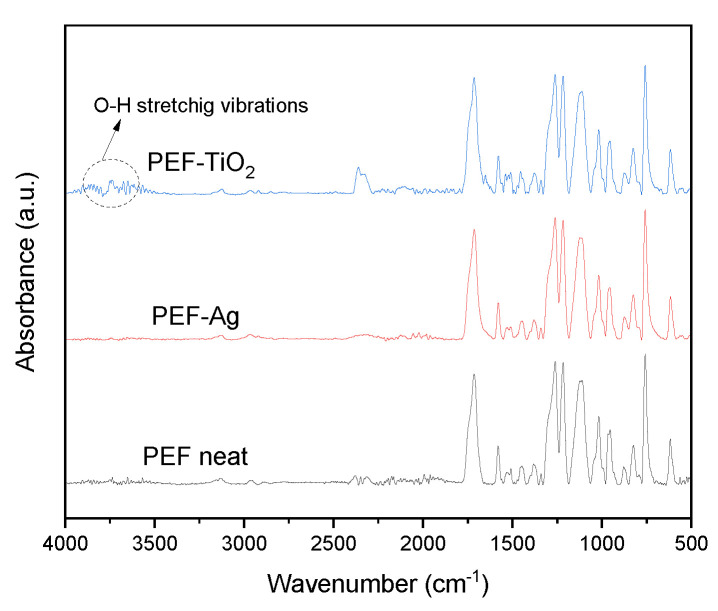
ATR–FTIR spectra of the neat PEF and respective nanocomposites.

The successful synthesis of PEF and respective nanocomposite materials was investigated using ATR-FTIR spectroscopy. Figure 3 displays the ATR spectra of PEF and PEF-based nanocomposite films. The recorded ATR spectra of all the samples are as follows: 3133–3156 cm^−1^ were related to C–H stretching of the furan ring, 2928–2936 cm^−1^ and 2846–2868 cm^−1^ were related to asymmetric and symmetric of C–H stretching vibrations, 1711–1727 cm^−1^ were linked to C–O stretching vibration, 1575–1581 cm^−1^ corresponds to aromatic C–C bending vibration, 1507–1512 cm^−1^ and 1451–1460 cm^−1^ were linked to C–H deformation and wagging vibrations, 1372–1384 cm^−1^ were related to C–H rocking vibrations, 1263–1267 cm^−1^ and 1222–1229 cm^−1^ were linked to C–O stretching vibrations, 1105–1114 cm^−1^ and 1009–1020 cm^−1^ were related to C–O–C vibrations of furan ring, 944–953 cm^−1^, 812–827 cm^−1^ and 760–766 cm^−1^ were linked to C–H out-of-plane deformation vibrations of furan ring, and 612–623 cm^−1^ corresponds to C–H bending vibrations [9,28].

Among the nanocomposites, PEF-TiO_2_ samples show tiny, sharp peaks around 3644–3708 cm^−1^, indicating O–H stretching vibrations, as seen in Figure 3. Due to the presence of –OH groups in TNPs, when TNPs were integrated into the PEF matrix, –OH groups were observed on nanocomposites [33,34,35].

### 3.4. ToF-SIMS Depth Profiling Analysis of the Nanocomposites

**Figure 4:**
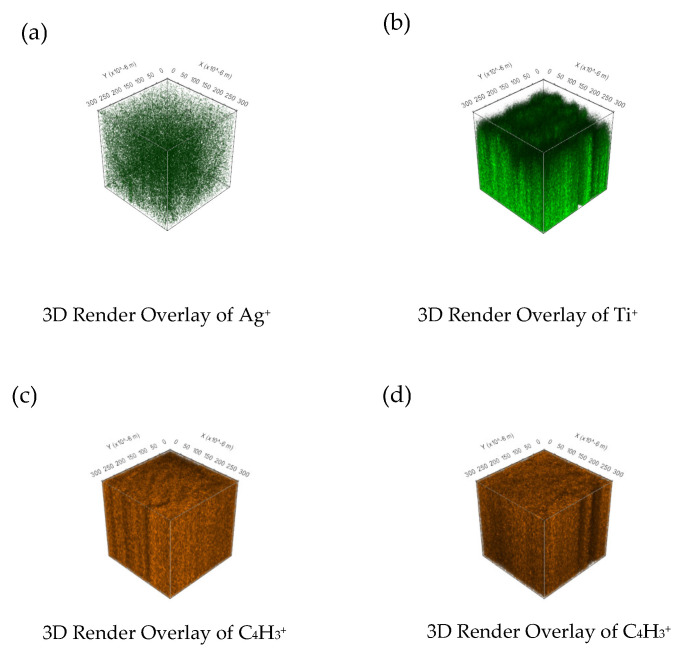
The spatial distribution of signals for (**a**) Ag^+^ in PEF-Ag nanocomposite and (**b**) Ti^+^ in PEF-TiO_2_ nanocomposite. The distribution of the C_4_H_3_^+^ signal representing the polymer matrix for (**c**) PEF-Ag nanocomposite and (**d**) PEF-TiO_2_ nanocomposite.

The ToF-SIMS approach opens up new avenues for material characterization by facilitating the analysis of 3D depth profiling of nanocomposites [36,37]. Figure 4 displays the distribution of Ag^+^ and Ti^+^ signals from AgNPs in the PEF-Ag nanocomposite and TNPs in the PEF-TiO_2_ nanocomposite. Ag^+^ ions were incorporated to improve the antimicrobial activity of PEF [18,24]. In our present work, only 1 wt.% of AgNPs were incorporated to avoid agglomeration of nanoparticles, and intense Ag^+^ signals were observed. Figure 4a confirms that AgNPs are homogeneously distributed into the PEF-Ag.

Like the PEF-Ag nanocomposite, intense Ti^+^ signals are observed even after incorporating a low amount (1 wt.%) of TNPs into the PEF matrix, intense Ti^+^ signals are observed. Figure 4b displays the 3D imaging of the PEF-TiO_2_ nanocomposite, where the Ti^+^ signals have been homogenously distributed into the PEF-TiO_2_ nanocomposite. Figure 4a,b display a low distribution of Ag^+^ and Ti^+^ signals on the surface of the nanocomposites, respectively. Figure 4c,d show the homogenous distribution of the C_4_H_3_^+^ signal for PEF-Ag nanocomposite and PEF-TiO_2_ nanocomposite, respectively.

### 3.5. Thermal Properties and Crystallinity

**Figure 5:**
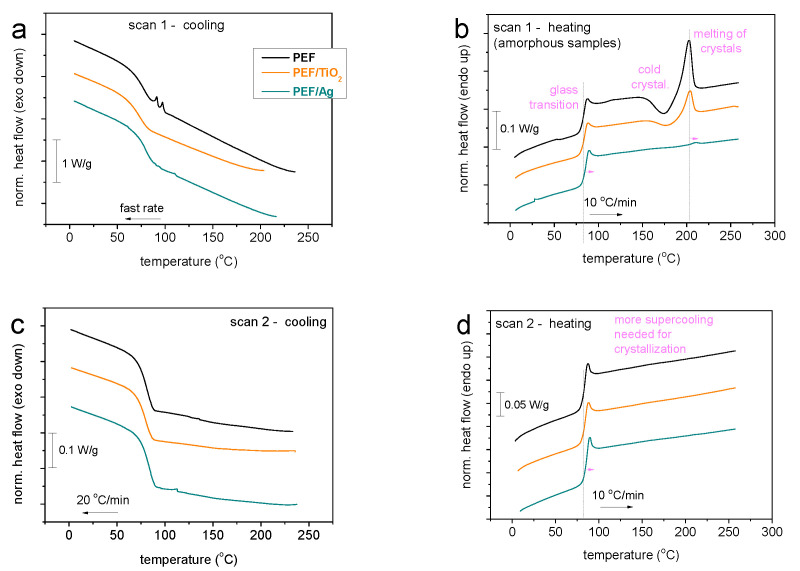
Comparative DSC thermograms for neat PEF and the two nanocomposites for both scans (**a**) during quenching, (**b**) subsequent heating after quenching, (**c**) cooling at 20 °C/min and (**d**) subsequent heating after cooling. The recorded heat flow is shown here upon normalization to the mass of the sample.

The thermal transitions and crystallization behavior of neat PEF and PEF-based nanocomposite materials were investigated using DSC analysis. The DSC curves for the standard scans 1 and 2 are presented in Figure 5. The following characteristic values were determined using DSC: melting temperature (*T_m_*), enthalpy (Δ*H_m_*), glass transition temperature (*T_g_*), heat capacity change during glass transition (Δ*c_p_*), crystallization and cold crystallization temperatures (*T_c_* and *T_cc_*), respectively, and a crystalline fraction (*X_c_*)* estimated by the enthalpies of crystallization and cold crystallization (Δ*H_c_* and Δ*H_cc_*, *X_c_* and *X_cc_*, respectively) were reported in Table 3. Upon erasing the thermal history, none of the systems exhibited crystallization during cooling. This is mainly expected for low-*M_n_* PEF, as reported by Papageorgiou et al. [38]. Thus, during the subsequent heating and until the glass transition event (step in Figure 5b,c), the samples remain amorphous. The characteristic temperature of glass transition, *T*_g_, was estimated by the half heat capacity, c_p_, increase, Δ*c*_p_. Thus, in the amorphous state, the *T*_g_ varies in the short range between 82–85 °C, increasing with the addition of nano inclusions. This shows the expected weak dependence from the *M_n_* [39] but may also be due to the interfacial interactions (constraints) between the polymer chains and the nanoparticles [40].

Interestingly, upon the fast cooling, both neat PEF and the nanocomposite materials exhibited weak exotherms arising from cold crystallization (at *T_cc_* 174–187 °C) and melting (at *T_m_* 203–210 °C) during heating. Furthermore, cold crystallization and melting are absent when the sample is subjected to slower cooling (Figure 5d). These findings demonstrated that significant supercooling was required to achieve crystal nucleation; however, as Figure 5 illustrates, nucleation and crystal growth are hindered by low *M_n_*. For the corresponding crystalline fractions (CF), *X_cc_*, are estimated between 0% and 6%. The characteristics values of neat PEF and nanocomposite are displayed in Table 3. Neat PEF exhibits relatively easier crystallizability, whereas the latter is hindered in the polymer nanocomposites. These effects are mainly expected, as has been discussed by Papageorgiou et al., they claim that the combination of the short polymer chains, the stiff backbone, and the carrying of the large-rigid furan ring are the primary causes of the inhibited nucleation in PEF [38]. Considering that *M_n_* mildly increases in the nanocomposites, the latter result provides another indirect indication for the existence of strong interfacial polymer/nanoparticle interactions [41]. The interfacial interactions should be actually present ab initio due to the in-situ nanocomposite preparation. To achieve an increased degree of crystallinity, we performed DSC measurements involving melt-crystallization annealing for a large period.

**Figure 6:**
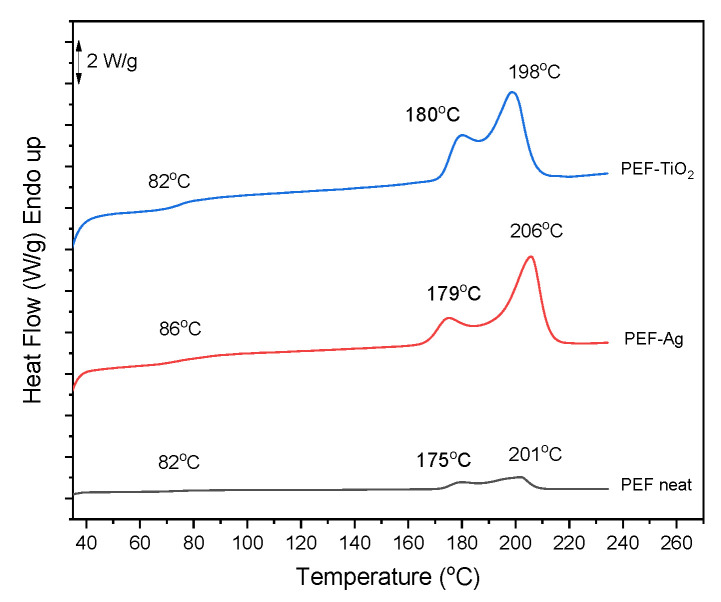
DSC scans of neat PEF and PEF-based nanocomposites after annealing (1st heating, rate 20 °C/min).

Figure 6 shows the calorimetric data from DSC scans after a one-hour annealing process (170 °C at 20 °C/min). The recrystallization–melting process causes the melting peak *T*_*m*2_ seen at higher temperatures [42,43,44]. In addition, *T_g_* of PEF-Ag tends to increase after annealing due to enhanced network connectivity, causing an increase in chain flexibility [45]. Finally, the increased flexibility of the polymer chain decreases the brittleness of the resulting polyester. The *T_g_* of PEF-TiO_2_ remains unchanged after annealing. PEF-based nanocomposite *X_c_^a^* elevated by more than 25%, showing that the recrystallization rate is unaffected by adding nanoparticles to the PEF matrix, as reported in Table 4 [9].

**Figure 7:**
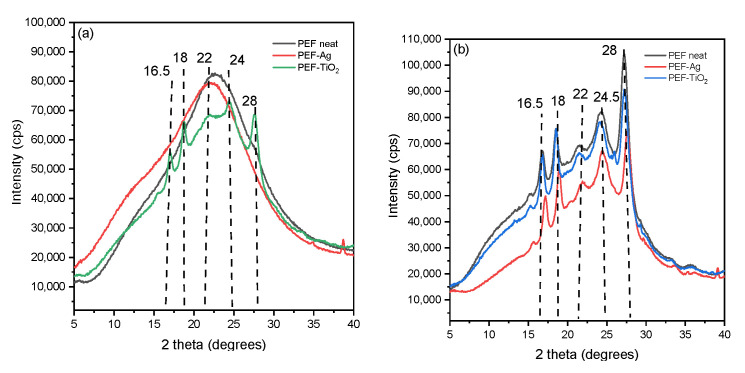
XRD patterns of (**a**) neat PEF and respective nanocomposites (amorphous) and (**b**) neat PEF and respective nanocomposites (annealed).

Figure 7a displays the XRD pattern of PEF neat and PEF-based nanocomposites. PEF-TiO_2_ nanocomposite exhibited the characteristic diffraction peaks of PEF at 2θ ≈ 16.5°, 2θ ≈ 18°, 2θ ≈ 22°, 2θ ≈ 24°, and 2θ ≈ 28°, which indicates the influence of crystallization after incorporation of TiO_2_ nanoparticle into the PEF matrix [46]. Table 4 displays the *X_c_^b^* that were determined from A_am_ and A_c_ utilizing Equation (4) and origin software (version 8.5.1). The degree of crystallinity of PEF-TiO_2_ was found to be 20.5%, which correlates with the DSC results. PEF-Ag nanocomposite and neat PEF displays an amorphous halo between 22.4° and 24.2°, indicating the samples’ amorphous nature.

Figure 7b exhibits the XRD pattern of annealed neat PEF and PEF nanocomposites. Results obtained from all the samples showed an increased *X_c_*. The diffraction peaks of PEF were observed at 2θ ≈ 16.5°, 2θ ≈ 18°, 2θ ≈ 22°, 2θ ≈ 24.5°, and 2θ ≈ 28°. The corresponding *X_c_^b^* values are displayed in Table 4. The degree of crystallinity calculated from the XRD patterns almost correlated with the DSC readings. Therefore, it is understood that adding nanoparticles to the PEF matrix does not affect crystallinity upon annealing.

### 3.6. Broadband Dielectric Spectroscopy

**Figure 8:**
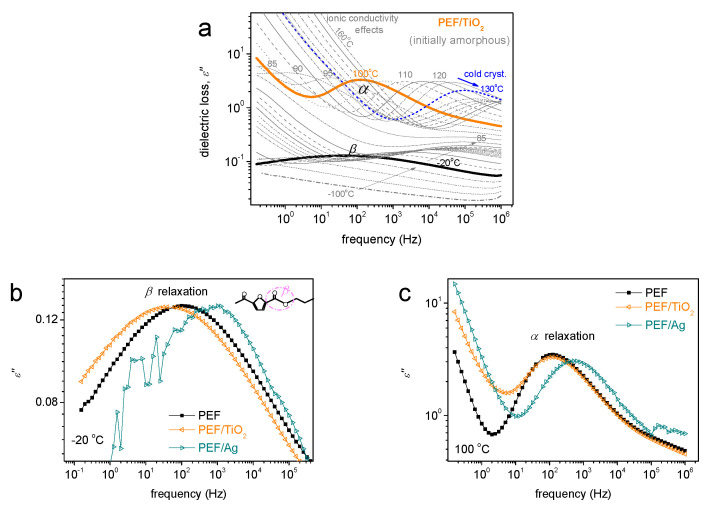
(**a**) The raw BDS data of amorphous PEF/TiO_2_ plotted against frequency for the recorded temperatures as the imaginary part of dielectric permittivity, ε″. (**b**,**c**) show comparative isothermal ε″(f) values for the two nanocomposites in relation to neat PEF at two chosen temperatures, (**b**) one below *T_g_* showing the local *β* process and (**c**) one above *T_g_* to present the effects imposed on the segmental α process.

BDS was employed for the study of molecular mobility. The latter is achieved by following the local and segmental mobility via their dipolar relaxation mechanisms. These relaxations are recorded as peaks of the imaginary part of dielectric permittivity against frequency, *ε*″*(f)*, for a wide range of frequencies and temperatures, i.e., below and well above *T_g_* [47]. With the temperature increasing, the relaxation *ε*″*(f)* peaks migrate toward higher frequencies, denoting the increased mobility due to the supplied thermal energy. This is actually the recording of molecular dynamics. Examples of the BDS results are shown in Figure 8. Basically, two types of molecular (dipolar) relaxations are recorded arising from the mobility of PEF, namely the local scale *β* and the segmental *α* relaxation. Both relaxations are present within the nanocomposites [48].

The local *β* relaxation, at lower temperatures, has been proposed to originate from crankshaft motions of the molecular group of the polymer related to the chemical link between the aromatic ring and the ester carbon aromatic ring [49,50]. At *T ≥ T_g_*, there is a significant signal increase, denoting the mobilization of the polymer chains. It is believed that during this mobilization, the dipole moments, being perpendicular to the polymer chain, are responsible for the recording of the strong α relaxation (dielectric analog of glass transition).

**Figure 9:**
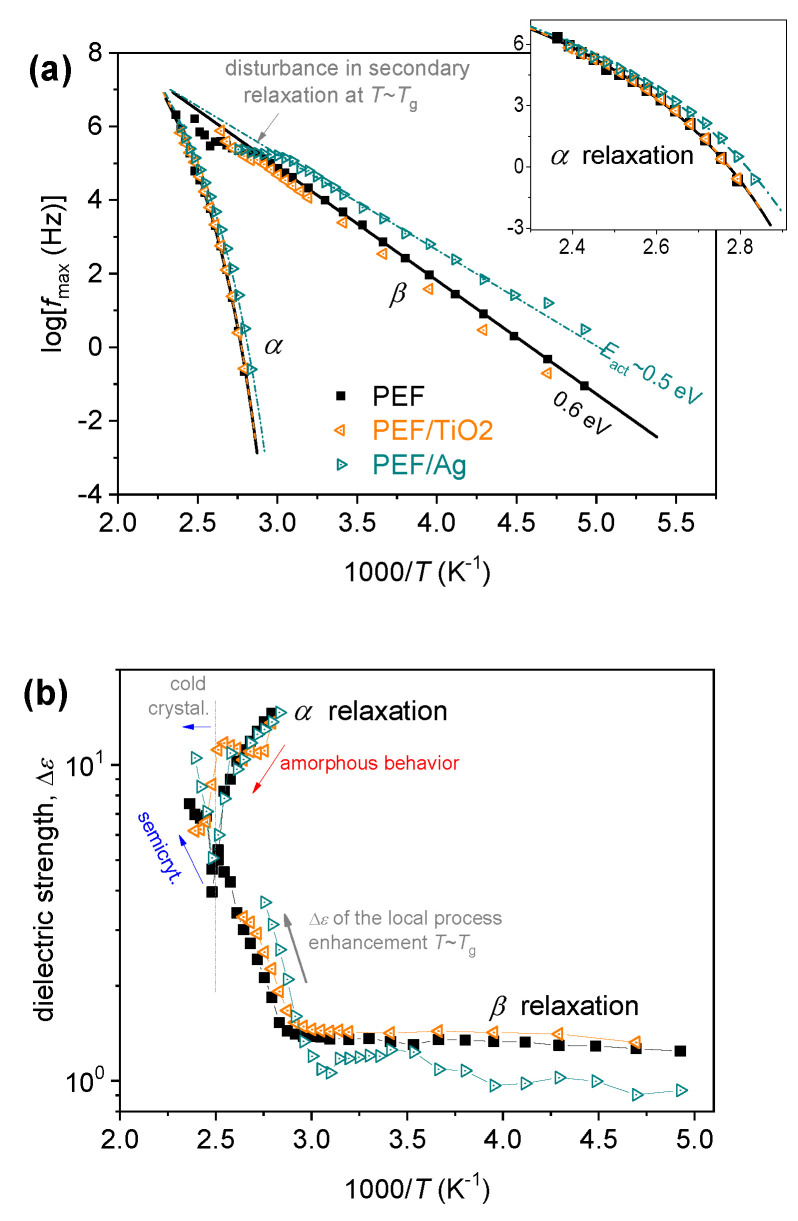
The dielectric activation map for all PEF-based systems, regarding (**a**) the reciprocal temperature dependences frequency maxima of the recorded relaxation peaks, logfmax, and (**b**) the same dependence of the corresponding dielectric strengths, Δ*ε*.

Following previous routes, a critical analysis in terms of fitting widely adopted model functions (Havriliak–Negami, Cole–Cole) was performed [51,52,53]. The results of this analysis are shown in Figure 9 in terms of timescale (relaxation frequency maxima vs. temperature, Figure 9a and dielectric strength, Δ*ε*, vs. temperature, known as the dielectric relaxation activation map.

In Figure 9a, the curved and straight lines connecting the experimental points have been added to represent the respective fitting of the Vogel–Tammann–Fulcher–Hesse and the Arrhenius equations, respectively, to *α* and *β* relaxation [51]. More precisely, the linear time scale behavior denotes that the activation energy is constant (independent from the temperature), which is the case for local dynamics. Contrariwise, the curved time scale trend denotes the cooperative character of the *α* relaxation.

The *β* relaxation of neat PEF exhibits a similar time scale in accordance with previous studies [51]. Interestingly, *β* is accelerated in PEF-Ag, accompanied by a slight decrease in its activation energy. Similarly, *α* relaxation shows an acceleration in PEF-Ag. The acceleration is, however, accompanied by a drop in its fragility index, *m_α_* (Table 5), the measure of cooperativity degree. Considering the *M_n_* increase for the sample, the effect is in the opposite direction, as shown in Table 5. So, to rationalize the combinatory effects of the structure on the segmental dynamics, a realistic scenario to explain these findings would involve the strong grafting of many PEF chains around the Ag nanoparticles, forming quite dense polymeric zones around the nanoparticles. Thus, a moderate increase in the free volume away from the nanoparticles would be expected, which can increase the interchain distances and, thus, lead to less or weaker chain–chain cooperativity. This is compatible with the acceleration of α relaxation and the mild decrease of *m_α_*.

Regarding PEF-TiO_2_, it is obvious that TiO_2_ nanoparticles impose fewer alternations on the polymer mobility, as the formation of less or weaker polymer-TiO_2_ interactions was expected [54]. This effect is compatible with the almost unchanged *M_n_* of neat PEF in the PEF-TiO_2_ systems. Based on previous findings on polymer nanocomposites, it is interesting to report that the larger size of TNPs compared to AgNPs results in less specific surface area (S_area_ in m^2^/g) being accessible for interaction with the polymer [55].

### 3.7. CA Analysis

**Figure 10:**
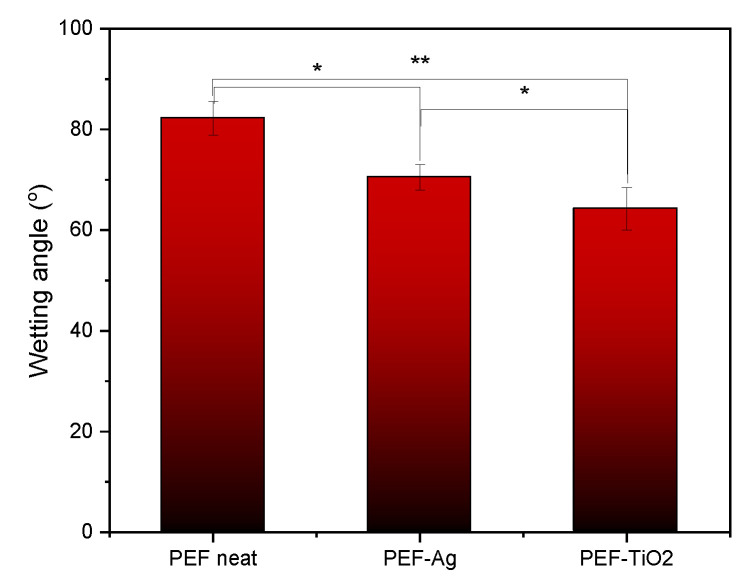
Contact angle values with standard deviation of neat PEF and respective nanocomposite films. One-way ANOVA. * *p* 0.01–0.05, ** 0.001 < *p* < 0.01.

Figure 10 displays the contact angle values of neat PEF and respective nanocomposites. The contact angle values of neat PEF were 82 ± 3.3 °C, PEF-Ag were 71 ± 2.5 °C, and PEF-TiO_2_ were 64 ± 4.2 °C. The average contact angle value of all PEF and respective nanocomposite films was <90 °, indicating its hydrophilic nature. The several factors affecting the contact values are due to the incorporation of inorganic nanoparticles, increased surface roughness, and altered surface energy.

The increase in surface roughness of PEF nanocomposites was observed through SEM images in Figure 2. In addition, the incorporation of metal nanoparticles can influence the surface energy of the polymer matrix, leading to changes in the contact angle. This high surface energy often makes metal nanoparticles highly reactive, which is advantageous for various applications such as catalysis, sensing, and biomedical applications. Thus, the surface free energy of a material increases and the contact angle decreases [56]. Furthermore, as shown by ATR-IR spectroscopy in Figure 3, the PEF-TiO_2_ nanocomposite exhibits a contact value of less than 70° because of the presence of hydrophilic hydroxyl groups (–OH). These hydroxyls can improve the interaction between the water molecules and the film’s surface while reducing the hydrophobicity [33,34,35,57].

### 3.8. Antimicrobial Studies

**Figure 11:**
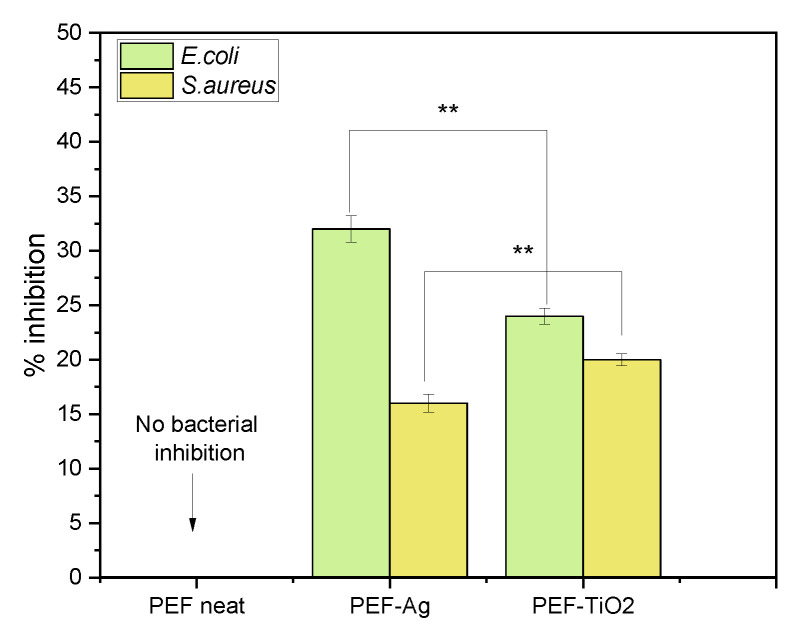
PEF and respective nanocomposites antimicrobial efficacy against *S. aureus* and *E. coli*. Multiple measurement two-way ANOVA: ** 0.001 < *p* < 0.01.

Figure 11 summarizes the antimicrobial activity of gram-positive bacteria (*S. aureus*) and gram-negative bacteria (*E. coli*) on neat PEF and PEF nanocomposite films after 6 h of incubation. Concerning neat PEF films, no inhibition percentage is recorded. However, incorporating AgNPs and TNPs as active agents moderately increases the inhibition % of *E. coli* and *S. aureus* after 6 h. It is due to the incorporation of very low wt.% of active agents into the PEF matrix, which shows a shallow distribution of NPs in the surface of nanocomposites as observed in ToF-SIMS images, Figure 4a,b. The literature shows that increasing the wt.% of active agents does not increase antimicrobial activity. On the other hand, it causes particle aggregation and clusters, which raise the surface roughness of the nanocomposites further [54].

Compared to neat PEF, the PEF-based nanocomposites show more than 15% inhibition against *S. aureus* and *E. coli*. The PEF-Ag film shows higher inhibition (32%) against gram-negative bacteria (*E. coli*) compared to gram-positive bacteria (*S. aureus*) with a 16% inhibition rate. While both types of bacteria can be susceptible to the antimicrobial activity of silver nanoparticles, there may be variations in their sensitivity due to differences in cell wall structure, membrane permeability, and other factors. Additionally, the concentration and availability of AgNPs on the material’s surface can also influence their inhibition percentage against different bacterial strains [58].

PEF-TiO_2_ nanocomposite film demonstrated 24% inhibition against *S. aureus* and 20% inhibition against *E.coli*. The increased inhibition rate compared to neat PEF is due to the large surface area and excellent morphology of TNPs in nature facilitating bacterial adsorption, accelerating the rate of bacterial inhibition [59]. Based on the literature, the % inhibition against *S. aureus* was more potent than that against *E. coli*, which may be due to the difference in structure and thickness of the membrane cell wall between *S. aureus* and *E. coli* [60,61]. Further research is needed to fully understand the mechanisms of action, optimize effectiveness, and mitigate potential risks of Ag and TiO_2_ nanoparticles incorporated into PEF for antimicrobial applications.

## 4. Conclusions

The PEF-based nanocomposites were fabricated using AgNPs and TNPs by the in-situ polymerization method with the objective of improving the thermal and antimicrobial properties of the nanocomposites. SEM micrographs exhibited that incorporating active agents led to the formation of a few aggregates on the surface of nanocomposites. ATR-FTIR results of PEF-TiO_2_ samples show O–H stretching vibrations due to the presence of -OH groups in TNPs. The distribution of nanoparticles into the polymer matrix was examined through ToF-SIMS analysis. The nanocomposites obtained a degree of crystallinity ranging between 0–6%, while the annealed samples showed an increase in crystallinity above 25% as noticed from DSC measurements. BDS studies enabled the complete mapping of local and segmental mobility for the first time, revealing an acceleration of segmental dynamics with suppressed chain cooperativity in the case of nanocomposites. These effects suggest both the implementation of strong interfacial interactions and increased free volume away from the nanoparticles. The contact angle values of PEF-based nanocomposites were decreased due to the incorporation of inorganic nanoparticles, increased surface roughness, and altered surface energy. Finally, both nanocomposites showed some initial inhibition against *E.coli* and *S. aureus*. PEF-Ag shows the highest inhibition (32%) against *E.coli*, and PEF-TiO_2_ exhibits the highest inhibition (24%) against *S. aureus*. Overall results show that the synthesized PEF-based nanocomposites could be used as secondary packaging materials with potential optimization.

## Figures and Tables

**Table 1:** Sample list, active agents, intrinsic viscosity, and molecular weight.

Sample	Active Agents (wt.%)	[*η*] (dL/g)	*M_n_* (g/mol)
PEF neat	-	0.43	8900
PEF-Ag	Silver nanoparticles (1)	0.47	10,200
PEF-TiO_2_	Titanium dioxide (1)	0.45	9600

**Table 2:** Color calorimetric measurements of neat PEF and respective nanocomposites.

Sample	L*	a*	b*	c*	h°	R	K/S
PEF neat	91.02	−2.17	7.78	8.57	108.60	47.14 (400 nm)	0.4
PEF-Ag	61.67	4.12	25.44	25.56	81.78	10.76 (400 nm)	3.7
PEF-TiO_2_	73.88	6.08	27.15	23.12	76.25	14.63 (400 nm)	2.5

**Table 3:** Characteristic values are determined through DSC measurements.

		Melt-Fast Cooled *Scan 1*						Melt-Slow Cooled *Scan 2*					
Sample	*M*_n_ (g/mol)	*T*_g_ (^o^C)	Δ*c*_p_ (J/g∙K)	*T*_cc_ (^o^C)	*X*_cc_ (%)	*T*_m_ (^o^C)	*X*_m_ (%)	*T*_c_ (^o^C)	*X*_c_ (%)	*T*_g_ (^o^C)	Δ*c*_p_ (J/g∙K)	*T*_m_ (^o^C)	Δ*H*_m_ (J/g)
PEF neat	8.9 k	82	0.46	174	5	203	6	-	0	82	0.46	-	0
PEF-TiO_2_	8.9 k	83	0.44	178	2	204	2	-	0	83	0.41	-	0
PEF-Ag	10.2 k	85	0.46	187	~0	210	~0	-	0	85	0.49	-	0

**Table 4:** Thermal characteristics from the DSC scan after annealing (170 °C, 1 h).

Samples	T_g_ (°C)	T_m1_ (°C)	T_m2_ (°C)	ΔH_m_ − ΔH_cc_(J/g)	*Xc *^a^ (%)	*Xc *^b^ (%)
PEF neat	82	175	201	40.18	29	31.5
PEF-Ag	86	179	206	37.90	27	28
PEF-TiO_2_	82	180	198	35.77	26	30

**^a^** From DSC data, using Equation (3). **^b^** From XRD data, using Equation (4).

**Table 5:** Estimated values regarding the segmental relaxation, namely, the dielectric glass transition temperature, *T_g_* diel, and the fragility index for the α relaxation, *m_α_*. For comparison, we have included the calorimetric data for amorphous *T_g_*.

		DSC *Scan 1*	BDS *Melted and Fast-Cooled*	
Sample	*M*_n_ (g/mol)	*T*_g_ (°C)	*T*_g, diel_ (°C)	*fragility index* *m*_α_
PEF neat	8.9k	82	75	100
PEF-TiO_2_	8.9k	83	75	101
PEF-Ag	10.2k	85	69	95

## Data Availability

Data are contained within the article.

## References

[1] Ludwicka K., Kaczmarek M., Białkowska A. (2020). Bacterial Nanocellulose—A Biobased Polymer for Active and Intelligent Food Packaging Applications: Recent Advances and Developments. Polymers.

[2] Mousavi Khaneghah A., Hashemi S.M.B., Limbo S. (2018). Antimicrobial Agents and Packaging Systems in Antimicrobial Active Food Packaging: An Overview of Approaches and Interactions. Food Bioprod. Process..

[3] Hamzehlou S., Aboudzadeh M.A. (2021). Special Issue on “Multifunctional Hybrid Materials Based on Polymers: Design and Performance”. Processes.

[4] Aboudzadeh M.A., Iturrospe A., Arbe A., Grzelczak M., Barroso-Bujans F. (2020). Cyclic Polyethylene Glycol as Nanoparticle Surface Ligand. ACS Macro Lett..

[5] de Jong E., Visser H.A., Dias A.S., Harvey C., Gruter G.-J.M. (2022). The Road to Bring FDCA and PEF to the Market. Polymers.

[6] Gabirondo E., Melendez-Rodriguez B., Arnal C., Lagaron J.M., Martínez de Ilarduya A., Sardon H., Torres-Giner S. (2021). Organocatalyzed Closed-Loop Chemical Recycling of Thermo-Compressed Films of Poly(Ethylene Furanoate). Polym. Chem..

[7] Zhu C., Yin J., Zhang Z., Shi F. (2022). Bio-Based Poly(Ethylene Furanoate)/ZnO Transparent Thin Films with Improved Water Vapor Barrier and Antibacterial Properties for Food Packaging Application. Mater. Res. Express.

[8] Zhu C., Ye D., Zhou T., Cui Y., Yin J. (2023). High-Antimicrobial Gallium-Doped Zinc Oxide Thin Films on Bio-Based Poly(Ethylene Furanoate) Substrates for Food Packaging Application. Membranes.

[9] Stanley J., Xanthopoulou E., Finšgar M., Zemljič L.F., Klonos P.A., Kyritsis A., Koltsakidis S., Tzetzis D., Lambropoulou D.A., Baciu D. (2023). Synthesis of Poly(Ethylene Furanoate) Based Nanocomposites by In Situ Polymerization with Enhanced Antibacterial Properties for Food Packaging Applications. Polymers.

[10] Stanley J., John A., Pušnik Črešnar K., Fras Zemljič L., Lambropoulou D.A., Bikiaris D.N. (2022). Active Agents Incorporated in Polymeric Substrates to Enhance Antibacterial and Antioxidant Properties in Food Packaging Applications. Macromol.

[11] Vilela C., Kurek M., Hayouka Z., Röcker B., Yildirim S., Antunes M.D.C., Nilsen-Nygaard J., Pettersen M.K., Freire C.S.R. (2018). A Concise Guide to Active Agents for Active Food Packaging. Trends Food Sci. Technol..

[12] Ashfaq A., Khursheed N., Fatima S., Anjum Z., Younis K. (2022). Application of Nanotechnology in Food Packaging: Pros and Cons. J. Agric. Food Res..

[13] Otoni C.G., Espitia P.J.P., Avena-Bustillos R.J., McHugh T.H. (2016). Trends in Antimicrobial Food Packaging Systems: Emitting Sachets and Absorbent Pads. Food Res. Int..

[14] Ahmed I., Lin H., Zou L., Brody A.L., Li Z., Qazi I.M., Pavase T.R., Lv L. (2017). A Comprehensive Review on the Application of Active Packaging Technologies to Muscle Foods. Food Control.

[15] Priyadarshi R., Roy S., Ghosh T., Biswas D., Rhim J.-W. (2022). Antimicrobial Nanofillers Reinforced Biopolymer Composite Films for Active Food Packaging Applications—A Review. Sustain. Mater. Technol..

[16] Qi Y., Xiang B., Tan W., Zhang J. (2017). Hydrophobic Surface Modification of TiO2 Nanoparticles for Production of Acrylonitrile-Styrene-Acrylate Terpolymer/TiO2 Composited Cool Materials. Appl. Surf. Sci..

[17] Mendes S.F., Costa C.M., Caparros C., Sencadas V., Lanceros-Méndez S. (2012). Effect of Filler Size and Concentration on the Structure and Properties of Poly(Vinylidene Fluoride)/BaTiO3 Nanocomposites. J. Mater. Sci..

[18] Kumar S., Shukla A., Baul P.P., Mitra A., Halder D. (2018). Biodegradable Hybrid Nanocomposites of Chitosan/Gelatin and Silver Nanoparticles for Active Food Packaging Applications. Food Packag. Shelf Life.

[19] Ediyilyam S., George B., Shankar S.S., Dennis T.T., Wacławek S., Černík M., Padil V.V.T. (2021). Chitosan/Gelatin/Silver Nanoparticles Composites Films for Biodegradable Food Packaging Applications. Polymers.

[20] Liu J., Ma Z., Liu Y., Zheng X., Pei Y., Tang K. (2022). Soluble Soybean Polysaccharide Films Containing In-Situ Generated Silver Nanoparticles for Antibacterial Food Packaging Applications. Food Packag. Shelf Life.

[21] Lara H.H., Garza-Treviño E.N., Ixtepan-Turrent L., Singh D.K. (2011). Silver Nanoparticles Are Broad-Spectrum Bactericidal and Virucidal Compounds. J. Nanobiotechnol..

[22] Azlin-Hasim S., Cruz-Romero M.C., Morris M.A., Cummins E., Kerry J.P. (2015). Effects of a Combination of Antimicrobial Silver Low Density Polyethylene Nanocomposite Films and Modified Atmosphere Packaging on the Shelf Life of Chicken Breast Fillets. Food Packag. Shelf Life.

[23] Chadha U., Bhardwaj P., Selvaraj S.K., Arasu K., Praveena S., Pavan A., Khanna M., Singh P., Singh S., Chakravorty A. (2022). Current Trends and Future Perspectives of Nanomaterials in Food Packaging Application. J. Nanomater..

[24] Othman S.H., Abd Salam N.R., Zainal N., Kadir Basha R., Talib R.A. (2014). Antimicrobial Activity of TiO_2_ Nanoparticle-Coated Film for Potential Food Packaging Applications. Int. J. Photoenergy.

[25] Siripatrawan U., Kaewklin P. (2018). Fabrication and Characterization of Chitosan-Titanium Dioxide Nanocomposite Film as Ethylene Scavenging and Antimicrobial Active Food Packaging. Food Hydrocoll..

[26] (2007). Plastics—Measurement of Antibacterial Activity on Plastics Surfaces.

[27] Kim E.S., Kim S.H., Lee C.H. (2010). Electrospinning of Polylactide Fibers Containing Silver Nanoparticles. Macromol. Res..

[28] Stanley J., Terzopoulou Z., Klonos P.A., Zamboulis A., Xanthopoulou E., Koltsakidis S., Tzetzis D., Zemljič L.F., Lambropoulou D.A., Kyritsis A. (2023). Effect of Monomer Type on the Synthesis and Properties of Poly(Ethylene Furanoate). Polymers.

[29] Muthulakshmi L., Rajini N., Nellaiah H., Kathiresan T., Jawaid M., Varada Rajulu A. (2017). Experimental Investigation of Cellulose/Silver Nanocomposites Using In Situ Generation Method. J. Polym. Environ..

[30] Alizadeh Sani M., Maleki M., Eghbaljoo-Gharehgheshlaghi H., Khezerlou A., Mohammadian E., Liu Q., Jafari S.M. (2022). Titanium Dioxide Nanoparticles as Multifunctional Surface-Active Materials for Smart/Active Nanocomposite Packaging Films. Adv. Colloid Interface Sci..

[31] Grothe J., Kaskel S., Leuteritz A. (2012). Nanocomposites and Hybrid Materials. Polymer Science: A Comprehensive Reference.

[32] Alizadeh-Sani M., Moghaddas Kia E., Ghasempour Z., Ehsani A. (2021). Preparation of Active Nanocomposite Film Consisting of Sodium Caseinate, ZnO Nanoparticles and Rosemary Essential Oil for Food Packaging Applications. J. Polym. Environ..

[33] Peng X., Ding E., Xue F. (2012). In Situ Synthesis of TiO2/Polyethylene Terephthalate Hybrid Nanocomposites at Low Temperature. Appl. Surf. Sci..

[34] Nanayakkara C.E., Larish W.A., Grassian V.H. (2014). Titanium Dioxide Nanoparticle Surface Reactivity with Atmospheric Gases, CO_2_, SO_2_, and NO_2_: Roles of Surface Hydroxyl Groups and Adsorbed Water in the Formation and Stability of Adsorbed Products. J. Phys. Chem. C.

[35] Wang Z., Wang X., Zhang J., Yu X., Wu Z. (2017). Influence of Surface Functional Groups on Deposition and Release of TiO_2_ Nanoparticles. Environ. Sci. Technol..

[36] Zou X., Chen K., Yao H., Chen C., Lu X., Ding P., Wang M., Hua X., Shan A. (2022). Chemical Reaction and Bonding Mechanism at the Polymer-Metal Interface. ACS Appl. Mater. Interfaces.

[37] Kravanja K.A., Finšgar M. (2021). Analytical Techniques for the Characterization of Bioactive Coatings for Orthopaedic Implants. Biomedicines.

[38] Papageorgiou G.Z., Papageorgiou D.G., Tsanaktsis V., Bikiaris D.N. (2015). Synthesis of the Bio-Based Polyester Poly(Propylene 2,5-Furan Dicarboxylate). Comparison of Thermal Behavior and Solid State Structure with Its Terephthalate and Naphthalate Homologues. Polymer.

[39] Klonos P.A., Papadopoulos L., Papageorgiou G.Z., Kyritsis A., Pissis P., Bikiaris D.N. (2020). Interfacial Interactions, Crystallization, and Molecular Dynamics of Renewable Poly(Propylene Furanoate) *In Situ* Filled with Initial and Surface Functionalized Carbon Nanotubes and Graphene Oxide. J. Phys. Chem. C.

[40] Sargsyan A., Tonoyan A., Davtyan S., Schick C. (2007). The Amount of Immobilized Polymer in PMMA SiO_2_ Nanocomposites Determined from Calorimetric Data. Eur. Polym. J..

[41] Terzopoulou Z., Klonos P.A., Kyritsis A., Tziolas A., Avgeropoulos A., Papageorgiou G.Z., Bikiaris D.N. (2019). Interfacial interactions, crystallization and molecular mobility in nanocomposites of poly(lactic acid) filled with new hybrid inclusions based on graphene oxide and silica nanoparticles. Polymer.

[42] Stoclet G., Arias A., Yeniad B., De Vos S. (2019). Relationships between Crystalline Structure and the Thermal Behavior of Poly(Ethylene 2,5-furandicarboxylate): An in Situ Simultaneous SAXS-WAXS Study. Polym. Eng. Sci..

[43] Mankar S.V., Garcia Gonzalez M.N., Warlin N., Valsange N.G., Rehnberg N., Lundmark S., Jannasch P., Zhang B. (2019). Synthesis, Life Cycle Assessment, and Polymerization of a Vanillin-Based Spirocyclic Diol toward Polyesters with Increased Glass-Transition Temperature. ACS Sustain. Chem. Eng..

[44] Righetti M.C., Marchese P., Vannini M., Celli A., Tricoli F., Lorenzetti C. (2019). Temperature-Induced Polymorphism in Bio-Based Poly(Propylene 2,5-Furandicarboxylate). Thermochim. Acta.

[45] Kaur A., Khanna A., Kaur A., Hirdesh, Gonzàlez-Barriuso M., Gonzàlez F. (2018). Effects of Annealing on Density, Glass Transition Temperature and Structure of Tellurite, Silicate and Borate Glasses. J. Non-Cryst. Solids.

[46] Stoclet G., Gobius du Sart G., Yeniad B., de Vos S., Lefebvre J.M. (2015). Isothermal Crystallization and Structural Characterization of Poly(Ethylene-2,5-Furanoate). Polymer.

[47] Kourtidou D., Klonos P.A., Papadopoulos L., Kyritsis A., Bikiaris D.N., Chrissafis K. (2021). Molecular Mobility and Crystallization of Renewable Poly(Ethylene Furanoate) *in Situ* Filled with Carbon Nanotubes and Graphene Nanoparticles. Soft Matter.

[48] Schönhals A., Szymoniak P. (2022). Dynamics of Composite Materials.

[49] Genovese L., Soccio M., Lotti N., Munari A., Szymczyk A., Paszkiewicz S., Linares A., Nogales A., Ezquerra T.A. (2018). Effect of Chemical Structure on the Subglass Relaxation Dynamics of Biobased Polyesters as Revealed by Dielectric Spectroscopy: 2,5-Furandicarboxylic Acid *vs. Trans* -1,4-Cyclohexanedicarboxylic Acid. Phys. Chem. Chem. Phys..

[50] Soccio M., Martínez-Tong D.E., Guidotti G., Robles-Hernández B., Munari A., Lotti N., Alegria A. (2020). Broadband Dielectric Spectroscopy Study of Biobased Poly(Alkylene 2,5-Furanoate)s’ Molecular Dynamics. Polymers.

[51] Bikiaris N.D., Klonos P.A., Ioannidis R.O., Saranti P., Barmpalexis P., Kyritsis A. (2024). Molecular mobility and thermal transitions study in renewable PLA-polyols star-like copolymers. Polymer.

[52] Xing K., Tress M., Cao P.-F., Fan F., Cheng S., Saito T., Sokolov A.P. (2018). The Role of Chain-End Association Lifetime in Segmental and Chain Dynamics of Telechelic Polymers. Macromolecules.

[53] Kremer F., Schönhals A. (2003). Broadband Dielectric Spectroscopy.

[54] Črešnar K.P., Aulova A., Bikiaris D.N., Lambropoulou D., Kuzmič K., Zemljič L.F. (2021). Incorporation of Metal-based Nanoadditives into the Pla Matrix: Effect of Surface Properties on Antibacterial Activity and Mechanical Performance of Pla Nanoadditive Films. Molecules.

[55] Klonos P., Kulyk K., Borysenko M.V., Gun’ko V.M., Kyritsis A., Pissis P. (2016). Effects of Molecular Weight below the Entanglement Threshold on Interfacial Nanoparticles/Polymer Dynamics. Macromolecules.

[56] Prakash J., Pivin J.C., Swart H.C. (2015). Noble Metal Nanoparticles Embedding into Polymeric Materials: From Fundamentals to Applications. Adv. Colloid Interface Sci..

[57] Zheng W., Sun C., Bai B. (2017). Molecular Dynamics Study on the Effect of Surface Hydroxyl Groups on Three-Phase Wettability in Oil-Water-Graphite Systems. Polymers.

[58] Lee J.H., Jeong D., Kanmani P. (2019). Study on Physical and Mechanical Properties of the Biopolymer/Silver Based Active Nanocomposite Films with Antimicrobial Activity. Carbohydr. Polym..

[59] Archana D., Singh B.K., Dutta J., Dutta P.K. (2013). In Vivo Evaluation of Chitosan–PVP–Titanium Dioxide Nanocomposite as Wound Dressing Material. Carbohydr. Polym..

[60] López de Dicastillo C., Guerrero Correa M., Martínez F.B., Streitt C., José Galotto M. (2021). Antimicrobial Effect of Titanium Dioxide Nanoparticles. Antimicrobial Resistance—A One Health Perspective.

[61] Xing Y., Li X., Zhang L., Xu Q., Che Z., Li W., Bai Y., Li K. (2012). Effect of TiO2 Nanoparticles on the Antibacterial and Physical Properties of Polyethylene-Based Film. Prog. Org. Coat..

